# Supplementary material for: Predictors of stent dysfunction in patients with bilateral metal stents for malignant hilar obstruction
Source: PLoS One. 2021 Mar 29;16(3):e0249096. doi: 10.1371/journal.pone.0249096 (PMC8007062; doi:10.1371/journal.pone.0249096)
Supplement: S1 File — (DOCX) [file pone.0249096.s001.docx]

**Supporting information method**

Data with patients who underwent plastic stent insertion for unresectable malignant hilar stricture were collected between March 2015 and June 2109. The data was compared with bilateral SEMS group insertion using Niti-S Large Cell D® (Taewoong medical, Gimpo, Korea) between September 2017 and February 2019 at Asan medical center and Kyung Hee University Hospital. All data was analyazed retrospectively. Descriptive statistics including mean, standard deviation, percentage were calculated. We estimated cumulative stent dysfunction using the Kaplan–Meier method. All reported P‐values were two‐sided, and a P‐value of less than 0.05 was considered to indicate statistical significance. Data were analyzed by using R program Ver. 3.5.3. (R foundation for Statistical Computing, Vienna, Austria, http://www.R-project.org).

**S1 table. Baseline characteristics of patients.**

| Patient related characteristics | Metal stent (n=87) | Plastic stent* (n=77) | P-value |
| --- | --- | --- | --- |
| Age, y | 67.4 ± 10.7 | 66.2 ± 12.1 | 0.529 |
| Male | 49 (56.3) | 38 (49.4) | 0.462 |
| Diagnosis |  |  | 0.080 |
| Cholangiocarcinoma (CCC), n | 69 (79.3) | 52 (67.5) |  |
| Gallbladder cancer | 9 (10.3) | 18 (23.4) |  |
| Others^￥^ | 9 (10.3) | 7 ( 9.1) |  |
| Bismuth type |  |  | 0.026 |
| Type II | 12 (13.8) | 24 (31.2) |  |
| Type III | 26 (29.9) | 20 (26.0) |  |
| Type IV | 49 (56.3) | 33 (42.9) |  |
| Procedure-related Characteristics |  |  |  |
| Cholangitis before stent insertion | 36 (41.4) | 30 (39.0) | 0.876 |
| Laboratory examination |  |  |  |
| Alkaline phosphatase | 481.9 ± 313.7 | 293.2 ± 196.6 | < 0.001 |
| Bilirubin | 6.5 ± 6.3 | 5.5 ± 4.5 | 0.221 |
| Following therapy |  |  |  |
| Chemotherapy | 49 (56.3) | 46 (59.7) | 0.776 |
| Radiotherapy | 17 (19.5) | 9 (11.7) | 0.246 |
| Outcomes of procedures |  |  |  |
| Clinical success, n (%) | 83 (95.4) | 68 (89.5) | 0.252 |
| Stent dysfunction, n (%) | 42 (48.3) | 44 (57.1) | 0.328 |
| Mean patency, days | 156.4 ± 116.6 | 80.4 ± 102.4 | < 0.001 |

All values were presented as mean ± standard deviation, number (%).

* Plastic stent group refers to patients with unresectable malignant hilar stricture who underwent bilateral plastic stent insertion (n=55) and unilateral stent insertion (n=22).

^￥^ Others in plastic stent group include 3 pancreatic, 1 ovarian, 1 gastric, 1 colon, 1 endometrial cancer.

**S1 Fig. Kaplan-Meier curve showing the cumulative stent patency.**


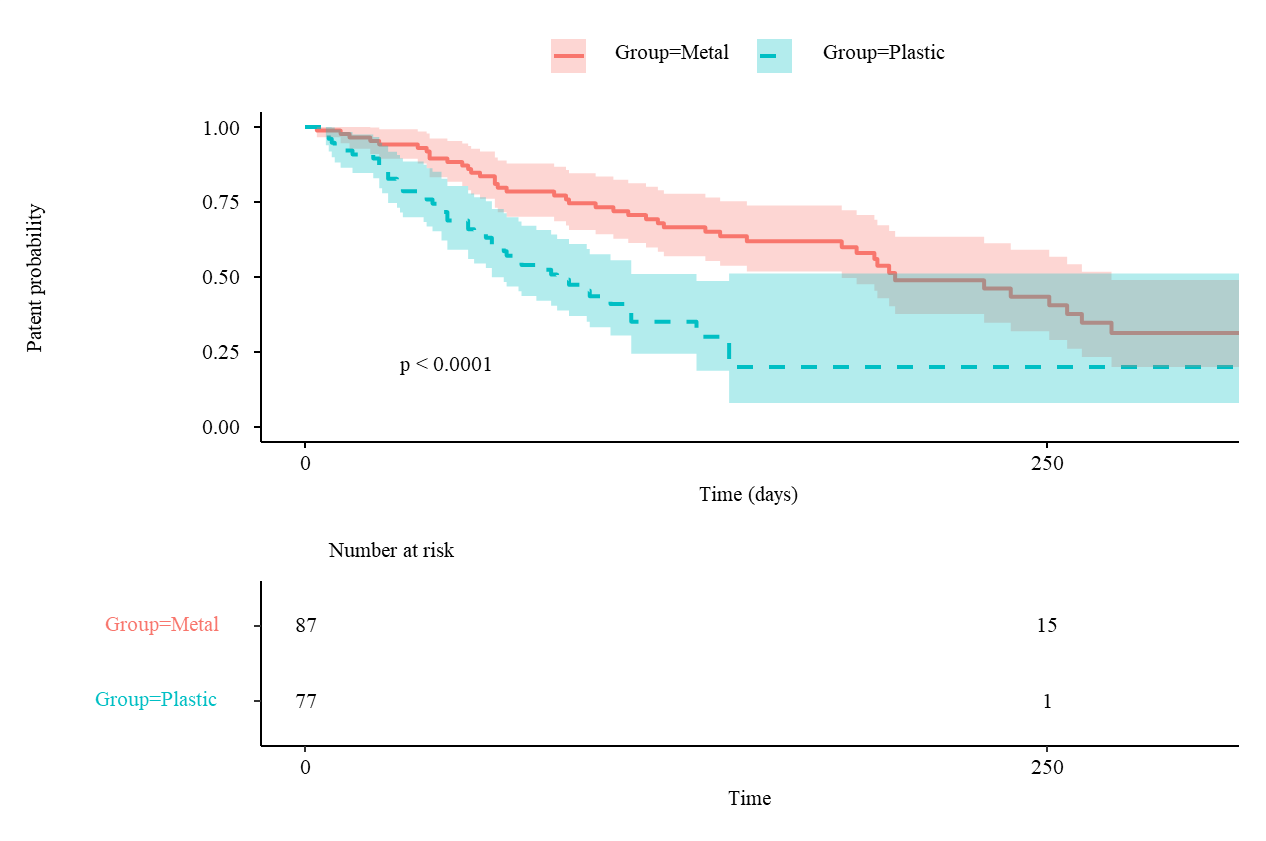


S1 Fig. legend.

Metal stent group showed longer patency compared to plastic stent group (HR 2.54, 95%CI: 1.600-4.026, P<0.0001)
