# Supplementary material for: Predictors of stent dysfunction in patients with bilateral metal stents for malignant hilar obstruction
Source: PLoS One. 2021 Mar 29;16(3):e0249096. doi: 10.1371/journal.pone.0249096 (PMC8007062; doi:10.1371/journal.pone.0249096)
Supplement: Attachment — Submitted filename: 4. Response to Reviewers.docx [file pone.0249096.s003.docx]

**Cover letter with point-by-point responses to reviewer comments**

**Manuscript number:** PONE-D-20-33082

**Manuscript title**: Predictors of stent dysfunction in patients with bilateral metal stents for malignant hilar obstruction

Dear Editor-in-Chief,

Thank you for reviewing our paper titled “Predictors of stent dysfunction in patients with bilateral metal stents for malignant hilar obstruction”. We appreciate the time you have taken to review our manuscript. We have made some corrections following your helpful comments, and we believe our paper has been much improved.

Therefore, we would like to resubmit this revised manuscript to “PLOS ONE” for publication. Point-by-point responses to the comments of the reviewers have also been prepared. All authors agreed to accept equal responsibility for the accuracy of the content of the paper. We hope the revised manuscript will better meet the requirements of “PLOS ONE” for publication and be finally accepted.

Data availability Statement

As there could be legal or ethical restriction of sharing our data by ethics committee, the data are available on request from the corresponding author (T.J.S.)

We thank you again for your thoughtful consideration.

Sincerely,

Tae Jun Song

Division of Gastroenterology

Asan Medical Center, University of Ulsan College of Medicine, Seoul, Korea

E-mail: drsong@amc.seoul.kr

**Point-by-point response**

**Response to reviewer #1**

1. I think study is interesting to show what this group expertise with double stenting. However, since it is a retrospective study, it would be very useful to add a group to compare plastic stents or PTC drains. I believe the group should have some historical patients with plastic stents / or PTC drains or common-bile duct single metal stents. One of these groups or all will increase the authors' thesis on the use of double metal stents.

**Response:** Thank you for your constructive comment. We agree with your suggestion. However, it was not easy to directly compare the outcomes of bilateral metal stents with those of plastic stents and/or PTC drains because of the heterogeneous clinical backgrounds and probability of selection bias of clinicians. Nevertheless, as suggested, our patients were compared with patients with plastic stents to confirm the longer patency of bilateral metal stent placement. We have reviewed the patients' data on unilateral or bilateral plastic stent placement for unresectable hilar malignant obstruction, and there were 77 cases of plastic stent insertion (55 were bilateral and 22 were unilateral). Baseline characteristics and clinical outcomes were analyzed, and patency was longer in the metal stent group than in the plastic stent group (HR 2.54; 95% CI: 1.600–4.026, P<0.0001). The limited number and heterogeneous baseline characteristics make comparisons difficult, and interpretation should be cautiously made. In retrospective and randomized controlled trials that compared SEMSs and plastic stents, SEMSs had higher technical and clinical success rates, required fewer reinterventions, and showed greater cost-effectiveness [1-3]. As this study’s aim was to report the clinical results of bilateral metal stent insertion, we think it would be better to include the data as supporting information.

In the **Discussion** section,

*Both plastic stents and SEMSs are commonly used for biliary drainage. Plastic stents are relatively cheap and can easily be exchanged. However, plastic stents have a high rate of obstruction due to their small diameter and thus must be replaced frequently. On the other hand, SEMSs are superior to plastic stents in terms of stent patency. In our retrospective analysis, SEMSs showed better patency compared with the patency of plastic stents (HR 2.54; 95% CI: 1.600–4.026, P<0.0001)* *(Supporting information).*

2. Minor: common abbreviation for cholangiocarcinoma is CCA not CCC. please use CCA throughout the manuscript.

**Response:** Thank you for pointing this out. We have revised CCC to CCA throughout the manuscript.

**Response to reviewer #2**

1. From the introduction, uncovered stents are more likely to be re-obstructed by tumor ingrowth. The current aimed to investigate factors associated with stent obstruction in a large-cell-type stent which is one type of uncovered stents. Based on this description, the reviewer expects to see the comparison of several parameters among large-cell-type and other types of stents. However, it is not present in the current study.

**Response:** Thank you for this constructive and important comment. Indeed, there are many different designs and types of metal stents, and depending on the nature/characteristics of the metal stents, they can affect patency. Although many types of metal stents are available for drainage of malignant hilar obstruction, we aimed to analyze factors that could affect the patency of bilateral stents in the study by using large cell-type stents. This study was a retrospective study, which limited the comparison of various types of metal stents. However, according to the comparative analysis of small cell-type and large cell-type metal stents for patients with malignant hilar obstruction in a retrospective study, stent patency according to the cell type did not differ [4]. The technical feasibility and functionality of closed and open cell-type SEMSs have not been evaluated. Usually, stents are selected according to the preference of the endoscopist and availability. For technical reasons, we performed bilateral stent replacement for malignant hilar obstruction in a stent-in-stent manner and thus preferred large cell-type metal stents. Therefore, retrospective comparisons of various stent types were not performed in this study. Nevertheless, the authors accept that the reviewer's comments are very important, so we have retrospectively compared and analyzed the patency of metal stents and plastic stents. The limited number and heterogeneous baseline characteristics make comparisons difficult, and interpretation should be cautiously made. As this study’s aim was to report the clinical results of bilateral metal stent insertion, we think it would be better to include the data as supporting information if you permit. Based on the important point and idea presented by the reviewer, we intend to conduct a prospective comparative study on stent patency, adverse event rate, and survival according to the stent replacement method (stent-in-stent vs. stent-by-stent) and type of metal stent (large cell-type vs. small cell-type). We have added the relevant information in the manuscript:

In the **Discussion** section,

*Finally, our study included patients with only large cell-type metal stents, which might limit the generalizability of the results. Patency may vary depending on the plastic stent, type of metal stent, and method of bilateral stent replacement. Our study aimed to investigate factors associated with stent obstruction with only large cell-type stents. In a retrospective study by Lee et al., there was no significant difference in stent patency according to the cell size of the metal stents (small cell-sized vs. large cell-sized stents, 42.9% vs. 45.5%, p = 0.086) [23]. Further investigation is necessary to compare several parameters among various types of stents and various methods of stent replacement.*

*23. Lee JM, Lee SH, Chung KH, Park JM, Paik WH, Woo SM, et al. Small cell- versus large cell-sized metal stent in endoscopic bilateral stent-in-stent placement for malignant hilar biliary obstruction. Dig Endosc. 2015;27:692-699. Epub 2015/02/25. doi: 10.1111/den.12466. PubMed PMID: 25708157.*

2. The study aimed to investigate factors associated with stent dysfunction in patients with malignant hilar obstruction. As suggested by the authors, the endoscopic placement of stents is currently a mainstay of its treatment in unresectable malignant hilar biliary obstruction. To improve the significance of this study, it is required to further analyze the difference between other therapies such as chemotherapies which are used for unresectable malignant hilar biliary obstruction and endoscopic placement of a large-cell-type stent.

**Response:** Thank you for your critical comment. As indicated in your comment, stent patency and survival will likely be affected by the use of local therapies, palliative care including chemotherapy/radiotherapy, and the development of functional stents and advanced techniques. The efficacy of local therapies such as photodynamic therapy (PDT) or radiofrequency ablation (RFA) in patients with advanced malignant hilar biliary obstruction is unclear; however, recent studies have reported benefits in terms of stent patency and survival [5-7]. Improving stent patency by PDT or RFA may allow complication-free survival and decrease reintervention for stent malfunction. Unfortunately, the current study is a retrospective analysis of several factors affecting the patency and survival of patients with large cell-type SEMSs, and further analysis was difficult due to the limited number of patients. Considering the need for further research as suggested by the reviewer, we will include more institutes to analyze the effects of various chemotherapies, local therapies, radiotherapies, etc. that may affect stent patency.

In the **Discussion** section,

*Fourth, the effects of local therapies such as intra-ductal radiofrequency ablation (RFA) and photodynamic therapy were not analyzed. Recently, it was found that bile duct carcinoma was associated with an increased frequency of intra-ductal RFA [22]. An analysis of the effects of local therapies on stent patency and survival is needed in the future.*

*22. Yang J, Wang J, Zhou H, Zhou Y, Wang Y, Jin H, et al. Efficacy and safety of endoscopic radiofrequency ablation for unresectable extrahepatic cholangiocarcinoma: A randomized trial. Endoscopy. 2018;50:751-760. Epub 2018/01/18. doi: 10.1055/s-0043-124870. PubMed PMID: 29342492.*

**References**

1.	Wagner HJ, Knyrim K, Vakil N, Klose KJ. Plastic endoprostheses versus metal stents in the palliative treatment of malignant hilar biliary obstruction. A prospective and randomized trial. Endoscopy. 1993;25:213-218.

2.	Sangchan A, Kongkasame W, Pugkhem A, Jenwitheesuk K, Mairiang P. Efficacy of metal and plastic stents in unresectable complex hilar cholangiocarcinoma: A randomized controlled trial. Gastrointest Endosc. 2012;76:93-99.

3.	Mukai T, Yasuda I, Nakashima M, et al. Metallic stents are more efficacious than plastic stents in unresectable malignant hilar biliary strictures: A randomized controlled trial. J Hepatobiliary Pancreat Sci. 2013;20:214-222.

4.	Lee JM, Lee SH, Chung KH, et al. Small cell- versus large cell-sized metal stent in endoscopic bilateral stent-in-stent placement for malignant hilar biliary obstruction. Dig Endosc. 2015;27:692-699.

5.	Sharaiha RZ, Natov N, Glockenberg KS, Widmer J, Gaidhane M, Kahaleh M. Comparison of metal stenting with radiofrequency ablation versus stenting alone for treating malignant biliary strictures: Is there an added benefit? Dig Dis Sci. 2014;59:3099-3102.

6.	Yang J, Wang J, Zhou H, et al. Efficacy and safety of endoscopic radiofrequency ablation for unresectable extrahepatic cholangiocarcinoma: A randomized trial. Endoscopy. 2018;50:751-760.

7.	Hong MJ, Cheon YK, Lee EJ, Lee TY, Shim CS. Long-term outcome of photodynamic therapy with systemic chemotherapy compared to photodynamic therapy alone in patients with advanced hilar cholangiocarcinoma. Gut Liver. 2014;8:318-323.
